# Association of Nonconcussive Repetitive Head Impacts and Intense Physical Activity With Levels of Phosphorylated Tau_181_ and Total Tau in Plasma of Young Elite Soccer Players

**DOI:** 10.1001/jamanetworkopen.2023.6101

**Published:** 2023-03-30

**Authors:** Martin Cente, Janka Perackova, Pavol Peracek, Marek Majdan, Igor Toth, Martin Mikulic, Jozef Hanes, Sara Porubska, Marian Spajdel, Barbora Kazickova, Igor Jurisica, Peter Filipcik

**Affiliations:** 1 Institute of Neuroimmunology, Slovak Academy of Sciences, Bratislava, Slovakia; 2 Department of Sport Science in Educology and Humanities, Faculty of Physical Education and Sport, Comenius University in Bratislava, Bratislava, Slovakia; 3 Department of Sports Games, Faculty of Physical Education and Sport, Comenius University in Bratislava, Bratislava, Slovakia; 4 Institute for Global Health and Epidemiology, Department of Public Health, Faculty of Health Sciences and Social Work, Trnava University, Trnava, Slovakia; 5 Department of Psychology, Faculty of Philosophy and Arts, Trnava University, Trnava, Slovakia; 6 Osteoarthritis Research Program, Division of Orthopedic Surgery, Schroeder Arthritis Institute and Data Science Discovery Centre for Chronic Diseases, Krembil Research Institute, University Health Network; 7 Departments of Medical Biophysics and Computer Science, and Faculty of Dentistry, University of Toronto, Ontario, Canada

## Abstract

**Importance:**

Head impacts resulting in traumatic brain injury (TBI) lead to the elevation of phosphorylated tau protein (p-tau_181_) in plasma. To our knowledge, this study is the first to investigate dynamics of p-tau_181_ levels and the ratio of p-tau_181_ to total tau in individuals after nonconcussive head impacts.

**Objective:**

To determine the association of repetitive low-intensity head impacts on p-tau_181_ and total tau protein levels in the plasma of young adult elite soccer players and assess the possible association of head impacts with focused attention and cognitive flexibility.

**Design, Setting, and Participants:**

In this cohort study, young elite soccer players performed intense physical activity with and without heading the ball. The study was conducted at a university facility in Slovakia from October 1, 2021, to May 31, 2022. Eligible participants were selected based on similarities in demographic variables, excluding those with a history of TBI.

**Main Outcomes and Measures:**

The primary study outcomes were the levels of total tau protein and p-tau_181_ in plasma samples and the cognitive status of the study participants.

**Results:**

A total of 37 male athletes participated in the study (mean [SD] age: exercise group, 21.6 [1.6] years; heading group, 21.2 [1.5] years). We found significantly elevated levels of total tau and p-tau_181_ in the plasma of soccer players 1 hour after physical exercise (tau, 1.4-fold; 95% CI, 1.2-1.5; *P* < .001; p-tau_181_, 1.4-fold; 95% CI, 1.3-1.5, *P* < .001) and repetitive head impacts (tau, 1.3-fold; 95% CI, 1.2-1.4; *P* < .001; p-tau_181_, 1.5-fold; 95% CI, 1.4-1.7 *P* < .001). The ratio of p-tau_181_ to tau was significantly higher 1 hour after exercise and heading training, and remained elevated specifically in the heading group even after 24 hours (1.2-fold; 95% CI, 1.1-1.3; *P* = .002). Performance in cognitive tests revealed a significant decline in focused attention and cognitive flexibility after physical exercise and heading training; physical exercise of higher intensity without heading training was associated with a greater negative cognitive performance than heading only.

**Conclusions and Relevance:**

In this cohort study of young elite soccer players, the elevation of p-tau_181_ and tau was observed after acute intense physical activity and nonconcussive repetitive head impacts. The increase of p-tau_181_ levels relative to tau after 24 hours indicated an acute enrichment of phosphorylated tau fraction in the periphery when compared with preimpact levels; an imbalance of tau proteins may have long-lasting consequences in the brain of head-impacted individuals.

## Key Points

**Question:**

What is the association of nonconcussive repetitive head impacts with plasma levels of phosphorylated and total tau proteins, and how might they be associated with cognitive outcomes in young elite soccer players?

**Findings:**

In a cohort study of 37 elite soccer players, a 1.5-fold increase of phosphorylated tau_181_ protein (p-tau_181_) in plasma after nonconcussive repetitive head impacts, and a 1.4-fold increase after intense physical activity was observed, while the elevation of the p-tau_181_-to-total-tau ratio 24 hours after training remained significant for nonconcussive repetitive head impacts. Performance in cognitive tests revealed a significant decline in focused attention and cognitive flexibility after the physical exercise and heading training.

**Meaning:**

These results suggest that the acute disbalance of the p-tau_181_ to total tau ratio may implicate emerging pathological processes in the early presymptomatic phase of neurological disorder.

## Teaser

This cohort study of elite youth soccer players in Slovakia examines the association of training drills with repeated nonconcussive heading with cognitive test performance compared with training with intense exercise only.

## Introduction

Neurodegeneration and dementia are major public health, medical, and societal problems, affecting 57.4 million patients in 2019 with projected trends increasing to 152.8 million cases by 2050.^1,2,3^ It is widely accepted that head impacts resulting in traumatic brain injury (TBI) are a significant risk factor for the development of sporadic tauopathies such as chronic traumatic encephalopathy (CTE), Alzheimer disease (AD), and other neurocognitive disorders.^4,5,6^ Mortality from neurodegenerative disorders was found to be elevated in former professional soccer players.^7^ In American football players, the severity of CTE is associated with the number of years played.^8^ The data on the consequences of low-intensity repetitive head impacts that are not followed by loss of consciousness (nonconcussive head impacts) are inconsistent. Therefore, it is not clear whether or not such mild head impacts are associated with neuropathology.^9^

Phosphorylated tau protein (p-tau) is a key pathogenic molecule that plays an important role in development of neurofibrillary degeneration.^10,11^ It was experimentally demonstrated that mild TBI leads to tau protein hyperphosphorylation, which induces the loss of intercellular connectivity and neuronal death.^12,13,14,15^ Tauopathies develop undetected before the onset of clinical signs and become apparent after a long period of latency.^16,17^ P-tau_181_ is considered a promising biomarker for early detection of AD, frontotemporal lobar degeneration, and TBI of all severities.^11,18,19,20,21,22,23^ While it has been suggested that cognitive tests should be an integral part of the holistic approach to assessment of TBI outcome,^24^ the cognitive consequences of minor, nonconcussive head impacts in context of p-tau quantification have not been widely studied.

To our knowledge, this is the first study investigating the effect of repetitive nonconcussive head impacts such as heading in soccer on the plasma level of p-tau_181_. We aimed to test the hypothesis that mild head impacts can lead to quantifiable changes in the plasma level of p-tau, and that these changes are accompanied by neurocognitive impairments at multiple time points after the impacts. We focused on quantifying plasma levels of total and phosphorylated tau in a highly uniform cohort, young male soccer players, before and after the physical training and regular heading training sessions.

## Methods

### Participants

Recruited participants were college soccer players at Comenius University who played competitions in the elite youth category U19 (ie, under age 19 years) and senior category outside the university environment in various Slovak soccer clubs. A total of 37 male young adults between ages 19 and 24 years participated in the study. Players underwent blood sampling and focused psychological tests prior to training sessions in order to establish the pretraining (reference) baseline in both domains. The ethical review board of the Comenius University in Bratislava, Faculty of Physical Education and Sport approved this study with human participants, and all involved athletes provided written informed consent prior to beginning the study. The study was conducted from October 1, 2021, to May 31, 2022, and followed the Strengthening the Reporting of Observational Studies in Epidemiology (STROBE) reporting guideline for cohort studies.

### Study Design

All athletes were evaluated for physical fitness by recording their individual heart rate using a monitor (Polar Electro Oy) at 5-second intervals. Individual performance of the athletes and the maximal heart rate were measured during a baseline training 7 days before the start of the study. The highest heart rate during the test was determined as maximal heart rate. The acquired data served as an indicator for designing the consecutive soccer training and heading sessions. This strategy aimed to maintain a high level of physical activity that was within the aerobic interval, represented by 70% to 75% of maximal heart rate. Furthermore, this target load intensity allowed simultaneous performance of aerobic soccer exercise and successful accomplishment of header tasks during heading training. Heart rate monitors were used during all training sessions. The players performed regular soccer training, including headers (mean [SD], 5.3 [4] headers per session, self-reported) 48 to 24 hours before baseline testing. The intensity of physical exertion within this time period was not recorded. Before and between blood collection time points, the players followed a resting regime for 24 hours. Baseline training (week 0) was followed by soccer training without heading the ball (week 1) and subsequent soccer training including the ball-heading drills (week 2). Further details on soccer training, heading training, impact force, video analysis, blood collection and processing, biochemical procedures and psychological testing are included in eMethods in Supplement 1.

### Outcomes

The primary study outcomes were the levels of total tau protein, p-tau_181_, and the ratio of p-tau_181_ to tau in plasma samples, as well as cognitive status (attention and flexibility) of the young elite soccer players. Study participants were assessed for primary outcomes at 3 time points, before (ie, from baseline to 0 hours) and after (1 hour and 24 hours) the 2 types of training, soccer exercise without heading the ball and soccer exercise including low-intensity repetitive headers.

### Statistical Analysis

Statistical analyses and figure processing were performed using GraphPad Prism version 9.3.1 (GraphPad Software). To correct for the basal, interindividual variability among the athletes, the plasma proteins concentration for each individual is expressed as a fold change at 1 hour and 24 hours from its own baseline. Group changes were visualized as mean fold change profiles with 95% CIs per training group. The mean fold change from baseline was compared in both training arms and at all time points (baseline to 0 hours, 1 hour, and 24 hours) using ANOVA for repeated measures considering the time (baseline, 1 hour, and 24 hour) as a within-participant factor and group (exercise, heading) as a between-participants factor, using Tukey post hoc tests. Correlation between the biomarkers, percentage of maximal heart rate, and number of headers was assessed with the Spearman correlation coefficients. Correlations and adjusted *P* values for multiple comparisons were considered significant at 2-sided *P* < .05 level.

## Results

### Demographic Information

This study included 37 college soccer players (all males of White European ancestry). The group of exercise athletes consisted of 32 players, the heading group included 28 players, 23 of whom also completed exercise training without headers. Participants of both training units did not differ significantly in demographic variables, such as mean age (exercise group, 21.6 [1.6] years vs heading plus exercise group, 21.2 [1.5] years), years of performing organized sport (14.2 [2.4] years vs 14.4 [2.1] years), weekly sports activity (407 [117] minutes vs 434 [110] minutes), body mass index (calculated as weight in kilograms divided by height in meters squared; 22.9 [1.8] vs 22.9 [1.6]), and self-reported history of TBI (0 in both groups) (Table 1). Individuals with a history of TBI were excluded.

**Table 1.** Characteristics of Study Participants and Training Parameters

Characteristics	Athletes, mean (SD)		*P* value
	Exercise (n = 32)	Heading (n = 28)	
Age, y			
Decimal	21.6 (1.6)	21.2 (1.5)	>.99
Sports^a^	14.2 (2.4)	14.4 (2.1)	>.99
Weekly sports activity, min	407 (117)	434 (110)	.21
BMI	22.9 (1.8)	22.9 (1.6)	>.99
History of TBI	0	0	
Training parameters			
Heart rate, bpm	138 (12)	131 (14)	.01
Maximal heart rate, bpm	189 (8)	188 (8)	>.99
Percentage at maximal heart rate	73 (5)	69 (6)	.01

Abbreviations: BMI, body mass index (calculated as weight in kilograms divided by height in meters squared); bpm, beats per minute; TBI, traumatic brain injury.

^a^ Sports age refers to years of performing organized sport activity.

### Training Parameters

**Figure 1.**
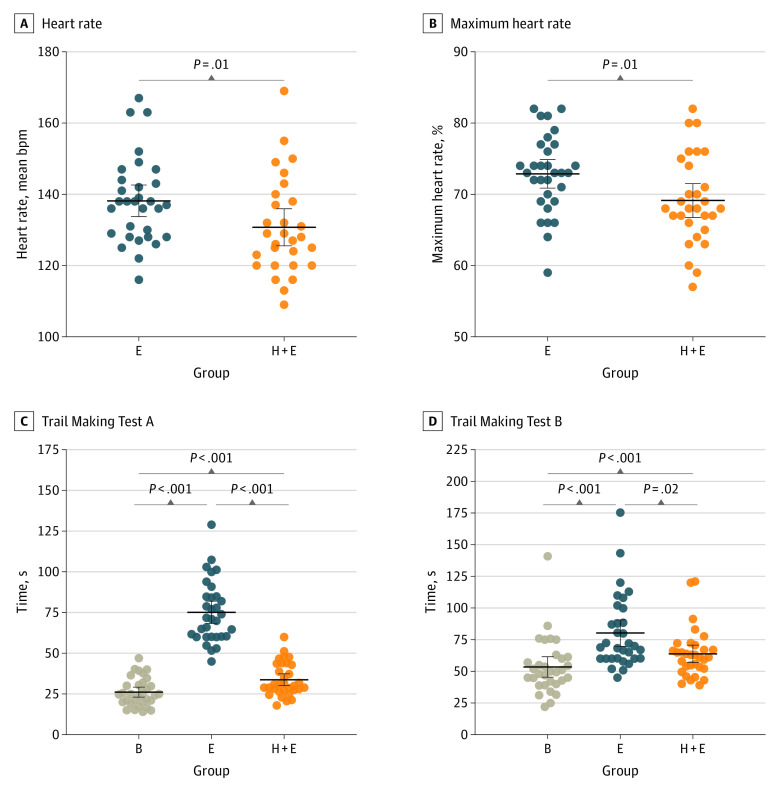
Physical Activity Parameters and Psychological Testing Heart rate monitoring during training units revealed that athletes performed exercise (E) with higher physical activity compared with headers with exercise (H + E), represented by the higher mean heart rate (panel A) and higher percentage of maximal heart rate (panel B). E and H + E were associated with decreases in the intensity and sustaining of attention of athletes when compared with baseline (B) as assessed by the Trail Making Test A (panel C). Both training sessions also showed negative outcomes on cognitive flexibility of athletes compared with the baseline (panel D). Psychological testing revealed that nonheading exercise was associated with lower test performance than exercise including heading training. Horizontal black lines denote the mean and 95% CI; bpm indicates beats per minute.

Physical performance of all athletes was monitored by heart rate monitors during both training sessions. Comparison of heart rate parameters revealed significantly higher mean heart rate of soccer players during the exercise without heading the ball when compared with the exercise including heading training (exercise group, 138 bpm; 95% CI, 134-143 bpm vs heading plus exercise group, 131 bpm; 95% CI, 126-136 bpm; *P* = .01) (Figure 1A). Both types of training also differed in the mean percentage of maximal heart rate that was higher during exercise without headers compared with heading training (73%; 95% CI, 71%-75% vs 69%; 95% CI, 67%-72%; *P* = .01), indicating more intensive physical exercise (Figure 1B).

The mean (SD) performed headers among players was 256 (35) (median [IQR], 251 [228-282]) (Table 2). The mean (SD) measured ball velocities during heading training were as follows: warm up heading (8.3 [0.1] m/s), heading in sitting position (8.3 [0.1] m/s), heading over defender (10.3 [0.1] m/s), square drill heading (11.1 [0.2] m/s), zig-zag exercise heading (13.9 [0.1] m/s), small-sided games heading (15.3 [0.2] m/s). Calculated impact forces together with average number of performed headers in specific heading exercises are summarized in Table 2.

**Table 2.** Characteristics of Heading Training

Heading training	No. of headers, mean (SD)	Impact force per 1 heading (SD), newtons
Warm up heading	19 (2)	187 (3)
Heading in sitting position	30 (1)	187 (2)
Heading over defender	28 (4)	232 (4)
Square drill heading	46 (4)	250 (4)
Zig-zag exercise heading	124 (33)	313 (3)
Small-sided games heading	9 (3)	344 (3)
Total headers	256 (35)	NA
Impact force, mean (Newtons)	NA	270 (51)

Abbreviation: NA, not applicable.

### Cognitive Outcomes

Neuropsychological examination of soccer players using both parts of the trail making test (TMT) identified significantly worse scores after exercise (mean TMT-A, 75.16 seconds; 95% CI, 68.30-82.01 seconds; *P* < .001; TMT-B, 80.26 seconds; 95% CI, 69.83-90.68 seconds; *P* < .001) and heading training including exercise (TMT-A, 33.76 seconds; 95% CI, 30.11-37.42 seconds; *P* < .001; TMT-B, 63.92 seconds; 95% CI, 56.99-70.84 seconds; *P* = .04) compared with baseline (TMT-A, 26.19 seconds; 95% CI, 23.06-29.31 seconds; TMT-B, 53.53 seconds; 95% CI, 45.55-61.50 seconds) testing (Table 3). Additionally, exercise without headers resulted in a significantly worse score in focused attention (exercise TMT-A, 75.16 seconds; 95% CI, 68.30-82.01 seconds vs heading plus exercise TMT-A, 33.76 seconds; 95% CI, 30.11-37.42 seconds; *P* < .001) and cognitive flexibility (exercise TMT-B, 80.26; 95% CI, 69.83-90.68 seconds vs heading plus exercise TMT-B, 63.92 seconds; 95% CI, 56.99-70.84 seconds; *P* = .02), even in contrast to training with heading (Figure 1C-D).

**Table 3.** Changes in Attention and Cognition After Exercise and Heading Training

Group	TMT-A			TMT-B		
	Mean (95% CI), seconds	*P* value		Mean (95% CI), seconds	*P* value	
		Vs baseline	Exercise without vs with heading		Vs baseline	Exercise with vs without heading
Baseline	26.19 (23.06-29.31)	NA	NA	53.53 (45.55-61.50)	NA	NA
Exercise	75.16 (68.3-82.01)	<.001	<.001	80.26 (69.83-90.68)	<.001	.02
Heading plus exercise	33.76 (30.11-37.42)	<.001		63.92 (56.99-70.84)	.04	

Abbreviations: NA, not applicable; TMT, trail making test.

### Changes in Circulating Plasma Neuroproteins After Exercise and Heading Training in Soccer Players

**Figure 2.**
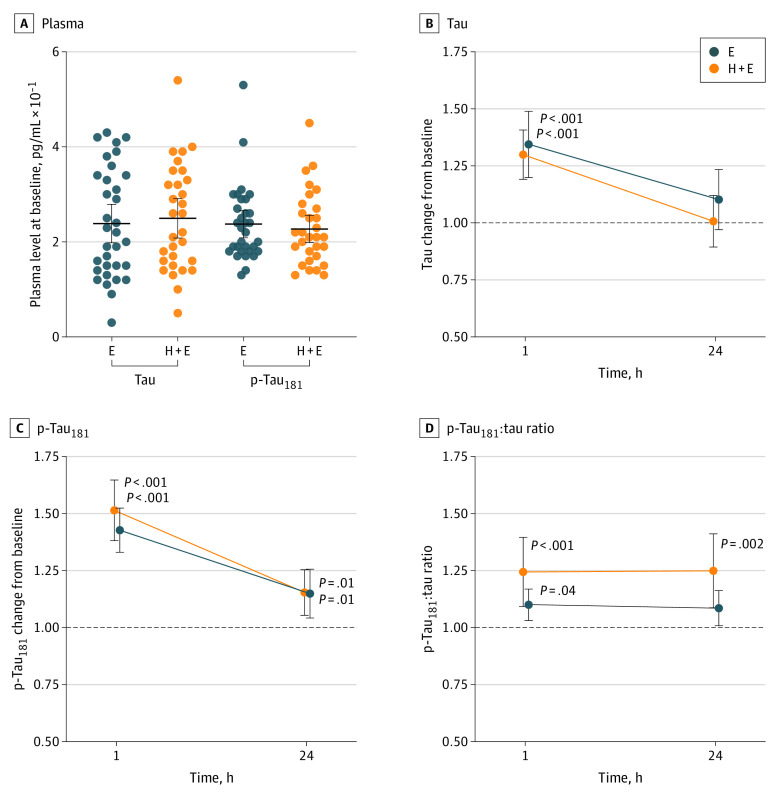
Tau and p-Tau_181_ Levels After Soccer Exercise (E) and Heading Plus Exercise (H + E) Analysis of total tau and phosphorylated tau protein at threonine 181 (p-tau_181_) in plasma revealed no difference between exercise without heading and exercise with heading training groups at baseline (panel A). Horizontal black lines denote the mean and 95% CI. Significant change in plasma tau from baseline was observed in athletes 1 hour after exercise and heading training. The elevated tau levels normalized to baseline (black dotted line) within the 24 hours (panel B). Significantly increased plasma p-tau_181_ levels were observed 1 hour after both types of training, and elevated levels were still detected after 24 hours (panel C). The ratio of p-tau_181_ to tau was significantly higher 1 hour after exercise and heading, and remained specifically elevated in the heading group even after 24 hours (panel D).

To elucidate the association of repetitive nonconcussive head impacts with neuronal markers, we performed a detailed analysis of total tau and p-tau_181_ in plasma of soccer players while accounting for the effect of physical activity. The mean plasma concentrations of total tau and p-tau_181_ examined at baseline were similar in both groups of athletes (Figure 2A). Quantitative digital enzyme-linked immunoassay identified similar significant change in plasma tau levels 1 hour after physical exercise (1.4-fold; 95% CI, 1.2-1.5; *P* < .001), and 1 hour after heading training including exercise (1.3-fold; 95% CI, 1.2-1.4; *P* < .001) when compared with baseline. The increased total tau levels in plasma of soccer players normalized to baseline within 24 hours (Figure 2B). The between-participant group factor was not significant, nor was the interaction between group and time. A similar increase, yet with a slightly higher magnitude of change, was observed for p-tau_181_ at 1-hour post-exercise (1.4-fold; 95% CI, 1.3-1.5; *P* < .001) and postheading including exercise (1.5-fold; 95% CI, 1.4-1.7; *P* < .001), respectively. An increased level of peripheral phosphorylated tau was identified even 24 hours posttraining (Figure 2C). Neither the effect of the group factor nor the interaction between the group and time factors were significant. We observed an increased plasma p-tau_181_-to-tau ratio in both groups at 1 hour postexercise (1.1-fold; 95% CI, 1.0-1.2; *P* = .04) and postheading including exercise (1.2-fold; 95% CI, 1.1-1.2; *P* < .001). However, the p-tau_181_-to-tau ratio remained significantly higher in heading group (1.2-fold; 95% CI, 1.1-1.3; *P* = .002) when compared with baseline even after 24 hours. The interaction between the group and time factors was not significant–the athletes performing soccer-related exercise without heading the ball showed a nonsignificant small decrease in p-tau_181_-to-tau ratio (1.1-fold; 95% CI, 1.0-1.2; *P* = .08) to pretraining level within 24 hours (Figure 2D).

Further analyses assessed the correlations between changes in total tau, p-tau_181_, and physical activity (percentage of maximal heart rate) in both groups of players. Percentage of maximal heart rate correlated with total tau (*r* = 0.6; *P* < .001) and p-tau_181_ (*r* = 0.6; *P* = .003) in the heading plus exercise group and with p-tau_181_ (*r* = 0.5; *P* = .007) in the exercise group at 1-hour time point. No correlation was observed between the number of low-intensity headers and changes in total tau (*r* = 0.05; *P* = .82) or p-tau_181_ (*r* = 0.06; *P* = .76) in plasma.

## Discussion

The main outcome of our study was the observation that tau protein p-tau_181_, which is a promising marker of neurodegeneration, was significantly increased by physical exercise and by heading the ball while playing soccer. While the fraction of total tau protein was shown to have increased in control athletes and athletes after concussion and TBI in several studies,^25,26^ we provide direct evidence that tau protein levels increased in the blood as a consequence of physical training, which is consistent with our previous observations.^27^ However, the significant increase in the level of p-tau_181_ relative to total tau in the plasma after regular physical soccer training and nonconcussive repetitive heading of the ball was unexpected, and to our knowledge is being reported for the first time. The finding was not expected in the exercise cohort, since the phosphorylation of tau protein is typically associated with TBI, and is one of the early diagnostic and prognostic biomarker candidates of AD dementia.^10,11,18,20^ However, it should be noted that the 1.5-fold increase of p-tau after mild heading training in this study was lower compared with the increase observed in mild or severe TBI cases.^20,26^ Increased p-tau_181_ even after 24 hours indicates longer persistence of phosphorylated tau in the brain. We emphasize the increase of tau and p-tau_181_ relative to tau after physical training as an important contributing factor to consider when evaluating tau levels in individuals experiencing sports-related head traumas. Importantly, our results provide valuable information to determine the analytical threshold for measuring the p-tau_181_-to-tau level in future TBI research and clinical practice. The observed correlations of physical exertion (ie, percentage of maximal heart rate) with tau and p-tau_181_ support the notion that there was an association between physical activity and total tau in the heading group and an association between physical activity and p-tau_181_ in both groups, respectively.

Both training units involved occasional player-to-player body contacts that are natural part of the game and are unavoidable in soccer. The body contacts occurring during ball tackling (eg, contact by hands and legs) were approximately the same in both types of training. We do not consider these kind of contacts as a major factor leading to the increase of tau and p-tau in this study.

Interestingly, the p-tau_181_-to-tau ratio remained significantly higher after exercise with mild repetitive head impacts when compared with preimpact levels. This also suggests that the amount of tau proteins is increased by a phosphorylated tau fraction, which may serve as a substrate for the accumulation of pathological forms of tau after a sufficient period of time.

Based on findings from Rubenstein et al,^20^ repetitive head impacts may lead to long-term pathological changes. We hypothesize that even the slight increase in the ratio of p-tau_181_ relative to tau that we observed after nonconcussive repetitive head impacts may represent a major difference for individuals playing contact sports vs other physical activities that do not involve routine head impacts. The possibility that even nonconcussive, very mild, multiple head impacts regularly repeated over time may lead to the development of neurocognitive disorders later in life cannot be excluded. Contact sports may therefore impose an increased health risk promoting the silent development of specific tauopathies (CTE, AD, etc) and progression of the pathology.^7,28^ Because the upregulation of p-tau could precede the development of the obvious pathological signs in behavior,^22^ we urge more precautions in contact sports, especially those in which, although very mild, repetitive head impacts are frequent, such as ball heading in soccer. It is especially important for at-risk groups, such as young adults and athletes with longer total exposure time.

Both types of soccer training analyzed in this study are within the same aerobic interval of physical performance, representing physiologically comparable groups. Nevertheless, the small difference in physical performance between the exercise and heading groups can be due to the higher level of concentration of athletes on the successful completion of ball-heading tasks, which resulted in a slightly lower level of physical activity in this specific group. Another surprising result of our study was finding that nonheading exercise was associated with a greater negative impact on cognition than heading training. Assuming the slightly higher physical performance in the exercise group, we hypothesize that even a small increase in physical activity was sufficient to induce the higher mental fatigue of athletes, which negatively affected the recorded cognitive parameters—focused attention and cognitive flexibility. The data were consistent with published evidence of decreased psychophysiological and cognitive responses related to mental fatigue after soccer,^29,30^ basketball,^31^ and table tennis^32^ training. Concerning the possible link between tau protein, intensity of physical activity, and cognition, the positive effect of physical activity in noncontact sports was associated with a slower rate of cognitive decline regardless of tau protein levels in a longitudinal perspective.^33^ As our study focused on acute effects, we did not observe any correlation between tau protein levels and cognitive parameters.

### Strengths and Limitations

The design of the study accounted for physical activity in studying the association of mild repetitive head impacts with cognitive function. Our experimental groups were relatively uniform, comprising athletes who did not differ significantly in demographic variables. In addition to the quantified parameters of physical activity, we also determined the number and intensity of head impacts, which increases the reproducibility of the study results.

Nevertheless, we acknowledge several limiting aspects of the study that need to be considered. In particular, we determined the level of phospho-fraction of tau protein by measuring only 1 phosphorylated epitope, threonine 181. More complex data should be collected, eg, quantifying other relevant tau populations with different phosphorylated epitopes such as tau_217_ and tau_231_. Consequently, it remains to be elucidated whether the increased level of p-tau_181_ is pathological or physiological. Results of this study might not extrapolate to the very acute effects of exercise (ie, less than 60 minutes) or repetitive head impacts on total tau and p-tau levels. Additionally, the impact forces listed were estimates that were not directly measured. Another limitation of the study was the number of participants; although this is a relatively highly uniform cohort, the results should be validated in a larger cohort and with additional follow-up time points. As this cannot be done by a single center or group, our study thus can stimulate many such studies in the near future. It will be also important to determine whether the same findings can be confirmed in female athletes. Based on our results, we acknowledge that an experimental group who take mild repetitive headers without any physical activity could help to further elucidate the complexity of physiological effects observed in this particular study.

## Conclusions

In this cohort study of young elite soccer players, we identified elevated levels of p-tau_181_, a biomarker of AD and TBI, as well as an increase in p-tau_181_-to-tau ratio, in the plasma of participants after physical exercise alone and after repetitive nonconcussive head impacts. The ratio of p-tau_181_ to tau, which remained elevated for a longer period of time in the head impact cohort, may indicate a prolonged molecular life span of phosphorylated tau protein in the brain of individuals with mild head impacts. These findings are potentially relevant for the assessment of the consequences of nonconcussive repetitive head impacts, such as heading in soccer, in association with an increased risk of developing neurodegenerative disorders later in life.

## Notes

Supplement 1.
eMethods.

eReferences.

Supplement 2.
Data Sharing Statement

## References

[1] Dewan MC, Rattani A, Gupta S, . Estimating the global incidence of traumatic brain injury. J Neurosurg. 2018;130(4):1080-1097. doi:10.3171/2017.10.JNS17352 29701556

[2] Traumatic Brain Injury GBD; GBD 2016 Traumatic Brain Injury and Spinal Cord Injury Collaborators. Global, regional, and national burden of traumatic brain injury and spinal cord injury, 1990-2016: a systematic analysis for the Global Burden of Disease Study 2016. Lancet Neurol. 2019;18(1):56-87. doi:10.1016/S1474-4422(18)30415-0 30497965 PMC6291456

[3] Dementia Forecasting Collaborators GBD; GBD 2019 Dementia Forecasting Collaborators. Estimation of the global prevalence of dementia in 2019 and forecasted prevalence in 2050: an analysis for the Global Burden of Disease Study 2019. Lancet Public Health. 2022;7(2):e105-e125. doi:10.1016/S2468-2667(21)00249-8 34998485 PMC8810394

[4] Baugh CM, Stamm JM, Riley DO, . Chronic traumatic encephalopathy: neurodegeneration following repetitive concussive and subconcussive brain trauma. Brain Imaging Behav. 2012;6(2):244-254. doi:10.1007/s11682-012-9164-5 22552850

[5] McKee AC, Stern RA, Nowinski CJ, . The spectrum of disease in chronic traumatic encephalopathy. Brain. 2013;136(Pt 1):43-64. doi:10.1093/brain/aws307 23208308 PMC3624697

[6] Sexton C, Snyder H, Beher D, . Current directions in tau research: highlights from Tau 2020. Alzheimers Dement. 2022;18(5):988-1007. doi:10.1002/alz.12452 34581500

[7] Mackay DF, Russell ER, Stewart K, MacLean JA, Pell JP, Stewart W. Neurodegenerative disease mortality among former professional soccer players. N Engl J Med. 2019;381(19):1801-1808. doi:10.1056/NEJMoa1908483 31633894 PMC8747032

[8] Mez J, Daneshvar DH, Abdolmohammadi B, . Duration of American football play and chronic traumatic encephalopathy. Ann Neurol. 2020;87(1):116-131. doi:10.1002/ana.25611 31589352 PMC6973077

[9] Mainwaring L, Ferdinand Pennock KM, Mylabathula S, Alavie BZ. Subconcussive head impacts in sport: a systematic review of the evidence. Int J Psychophysiol. 2018;132(Pt A):39-54. doi:10.1016/j.ijpsycho.2018.01.007 sport 29402530

[10] Guo T, Noble W, Hanger DP. Roles of tau protein in health and disease. Acta Neuropathol. 2017;133(5):665-704. doi:10.1007/s00401-017-1707-9 28386764 PMC5390006

[11] Wegmann S, Biernat J, Mandelkow E. A current view on Tau protein phosphorylation in Alzheimer’s disease. Curr Opin Neurobiol. 2021;69:131-138. doi:10.1016/j.conb.2021.03.003 33892381

[12] Smith DH, Kochanek PM, Rosi S, . Roadmap for advancing pre-clinical science in traumatic brain injury. J Neurotrauma. 2021;38(23):3204-3221. doi:10.1089/neu.2021.0094 34210174 PMC8820284

[13] Petersen A, Soderstrom M, Saha B, Sharma P. Animal models of traumatic brain injury: a review of pathophysiology to biomarkers and treatments. Exp Brain Res. 2021;239(10):2939-2950. doi:10.1007/s00221-021-06178-6 34324019

[14] Skotak M, Townsend MT, Ramarao KV, Chandra N. A comprehensive review of experimental rodent models of repeated blast TBI. Front Neurol. 2019;10:1015. doi:10.3389/fneur.2019.01015 31611839 PMC6776622

[15] Brett BL, Gardner RC, Godbout J, Dams-O’Connor K, Keene CD. Traumatic brain injury and risk of neurodegenerative disorder. Biol Psychiatry. 2022;91(5):498-507. doi:10.1016/j.biopsych.2021.05.025 34364650 PMC8636548

[16] Chung DC, Roemer S, Petrucelli L, Dickson DW. Cellular and pathological heterogeneity of primary tauopathies. Mol Neurodegener. 2021;16(1):57. doi:10.1186/s13024-021-00476-x 34425874 PMC8381569

[17] VanItallie TB. Traumatic brain injury (TBI) in collision sports: Possible mechanisms of transformation into chronic traumatic encephalopathy (CTE). Metabolism. 2019;100S:153943. doi:10.1016/j.metabol.2019.07.007 31610856

[18] Barthélemy NR, Li Y, Joseph-Mathurin N, ; Dominantly Inherited Alzheimer Network. A soluble phosphorylated tau signature links tau, amyloid and the evolution of stages of dominantly inherited Alzheimer’s disease. Nat Med. 2020;26(3):398-407. doi:10.1038/s41591-020-0781-z 32161412 PMC7309367

[19] Janelidze S, Mattsson N, Palmqvist S, . Plasma p-tau_181_ in Alzheimer’s disease: relationship to other biomarkers, differential diagnosis, neuropathology and longitudinal progression to Alzheimer’s dementia. Nat Med. 2020;26(3):379-386. doi:10.1038/s41591-020-0755-1 32123385

[20] Rubenstein R, Chang B, Yue JK, ; the TRACK-TBI Investigators. Comparing plasma phospho tau, total tau, and phospho tau-total tau ratio as acute and chronic traumatic brain injury biomarkers. JAMA Neurol. 2017;74(9):1063-1072. doi:10.1001/jamaneurol.2017.0655 28738126 PMC5710183

[21] Thijssen EH, La Joie R, Wolf A, ; Advancing Research and Treatment for Frontotemporal Lobar Degeneration (ARTFL) investigators. Diagnostic value of plasma phosphorylated tau181 in Alzheimer’s disease and frontotemporal lobar degeneration. Nat Med. 2020;26(3):387-397. doi:10.1038/s41591-020-0762-2 32123386 PMC7101073

[22] Palmqvist S, Tideman P, Cullen N, ; Alzheimer’s Disease Neuroimaging Initiative. Prediction of future Alzheimer’s disease dementia using plasma phospho-tau combined with other accessible measures. Nat Med. 2021;27(6):1034-1042. doi:10.1038/s41591-021-01348-z 34031605

[23] Shahim P, Politis A, van der Merwe A, . Time course and diagnostic utility of NfL, tau, GFAP, and UCH-L1 in subacute and chronic TBI. Neurology. 2020;95(6):e623-e636. doi:10.1212/WNL.0000000000009985 32641529 PMC7455355

[24] Maas AIR, Menon DK, Manley GT, ; InTBIR Participants and Investigators. Traumatic brain injury: progress and challenges in prevention, clinical care, and research. Lancet Neurol. 2022;21(11):1004-1060. doi:10.1016/S1474-4422(22)00309-X 36183712 PMC10427240

[25] Gill J, Merchant-Borna K, Jeromin A, Livingston W, Bazarian J. Acute plasma tau relates to prolonged return to play after concussion. Neurology. 2017;88(6):595-602. doi:10.1212/WNL.0000000000003587 28062722 PMC5304458

[26] Czeiter E, Amrein K, Gravesteijn BY, ; CENTER-TBI Participants and Investigators. Blood biomarkers on admission in acute traumatic brain injury: relations to severity, CT findings and care path in the CENTER-TBI study. EBioMedicine. 2020;56:102785. doi:10.1016/j.ebiom.2020.102785 32464528 PMC7251365

[27] Sandmo SB, Filipcik P, Cente M, . Neurofilament light and tau in serum after head-impact exposure in soccer. Brain Inj. 2020;34(5):602-609. doi:10.1080/02699052.2020.1725129 32096660

[28] Kriegel J, Papadopoulos Z, McKee AC. Chronic traumatic encephalopathy: is latency in symptom onset explained by tau propagation? Cold Spring Harb Perspect Med. 2018;8(2):a024059. doi:10.1101/cshperspect.a024059 28096246 PMC5793739

[29] Sun H, Soh KG, Roslan S, Wazir MRWN, Soh KL. Does mental fatigue affect skilled performance in athletes? a systematic review. PLoS One. 2021;16(10):e0258307. doi:10.1371/journal.pone.0258307 34648555 PMC8516214

[30] Nédélec M, McCall A, Carling C, Legall F, Berthoin S, Dupont G. Recovery in soccer: part I—post-match fatigue and time course of recovery. Sports Med. 2012;42(12):997-1015. doi:10.2165/11635270-000000000-00000 23046224

[31] Moreira A, Aoki MS, Franchini E, da Silva Machado DG, Paludo AC, Okano AH. Mental fatigue impairs technical performance and alters neuroendocrine and autonomic responses in elite young basketball players. Physiol Behav. 2018;196:112-118. doi:10.1016/j.physbeh.2018.08.015 30172721

[32] Le Mansec Y, Pageaux B, Nordez A, Dorel S, Jubeau M. Mental fatigue alters the speed and the accuracy of the ball in table tennis. J Sports Sci. 2018;36(23):2751-2759. doi:10.1080/02640414.2017.1418647 29260619

[33] Desai P, Evans D, Dhana K, . Longitudinal association of total tau concentrations and physical activity with cognitive decline in a population sample. JAMA Netw Open. 2021;4(8):e2120398. doi:10.1001/jamanetworkopen.2021.20398 34379124 PMC8358733

