# Supplementary material for: Association of Nonconcussive Repetitive Head Impacts and Intense Physical Activity With Levels of Phosphorylated Tau181 and Total Tau in Plasma of Young Elite Soccer Players
Source: JAMA Netw Open. 2023 Mar 30;6(3):e236101. doi: 10.1001/jamanetworkopen.2023.6101 (PMC10064253; doi:10.1001/jamanetworkopen.2023.6101)
Supplement: Supplement 1. — eMethods. eReferences. [file jamanetwopen-e236101-s001.pdf]

Supplemental Online Content

Cente M, Perackova J, Peracek P, et al. Association of nonconcussive repetitive head impacts and intense physical activity with levels of phosphorylated tau_{181} and total tau in plasma of young elite soccer players. *JAMA Netw Open.* 2023;6(3):e236101. doi:10.1001/jamanetworkopen.2023.6101

eMethods.

eReferences.

This supplemental material has been provided by the authors to give readers additional information about their work.

© 2023 Cente M et al. *JAMA Network Open.*

eMethods.

Soccer training

All training sessions during the experiment were planned and controlled by professional trainers ensuring consistency across athletes and sessions. The soccer training unit (hereafter referred to as exercise, abbreviated as "E") comprised a warm-up (22 min), two exercise blocks (58 min), and a final compensatory exercise (5 min) with a total duration of 85 min. The warm-up part of the training included running (7 min), followed by exercise without the ball (6 min) and exercise with the ball (9 min). The main part of the training unit consisted of 2 different small-sided games. Players were not allowed to do headers during the entire soccer training unit, nor were exposed to any kind of player-to-player head contacts.

Heading training including exercise

Heading training (abbreviated "H+E") followed a modified physical exercise protocol when compared to soccer training. All parts, including the warm-up and main training units contained the heading of the ball. The warm-up included two sets of three-meter hand passes of the ball to the head of a teammate. The main heading training included 5 blocks of heading exercise: 1. Heading after hands pass in a sitting position (sit with the spread legs) for three sets, each set with 10 attempts of heading; 2. Heading over the semi-active defender after hands pass in two sets of 15 attempts each; 3. Heading in the square for four sets, each set lasted 3 minutes followed by 30-second rest interval; 4. Heading in the zig-zag exercises for four sets, each set lasted 3 minutes with 30-second rest interval; 5. Five small-sided games (SSG) with a given pitch size (25x18 meters) and a rule that a goal can only be scored with a header. During the SSG, hand pass and a head pass were continuously repeated. Each small-sided game lasted 2 minutes followed by 2-minute rest interval. The total duration of the heading training was 85 min.

Impact force

To ensure higher reproducibility and to characterize the range of head impact intensities, ball impact forces were assessed, providing a quantitative estimate of head impacts in specific training sessions. The average impact force (F) for specific heading exercises was calculated using the formula:

$$F = \frac{vm}{t}$$

Assuming inputs (v) as incoming velocity (m/s), (m) mass of the ball (kg) and (t) impact duration (s) [34].
Incoming velocity of the ball in specific blocks during heading exercise was quantified using the radar LM600 (UNI-Trend Technology). The ball mass (0.45 kg) was considered as indicated by the manufacturer. Throughout the study, only officially tested FIFA Quality Pro marked balls (Adidas Uniforia League Pro) were used. The average time of impact duration (0.02 s) was adopted according to published data for ball size 5 and inflation pressure of 15 psi (1 bar) at the speed of 15 $m.s^{-1}$ [35], considering the velocity as a most important factor contributing to the peak impact force [36].
All the ball parameters listed are in accordance with the International Football Association Board 2020/21 Laws of the game, Law 2 [37].
The ball heading training represents a pool of low-intensity head impacts that correspond to the practice of heading techniques routinely performed in youth to adult categories in soccer. The average impact force of 270 *Newtons (N)* as quantified in this study is substantially lower compared to peak impact forces of soccer balls (1700 *N*) measured at higher speeds in the elite soccer league [35,38,39]. High-intensity head impacts, which are typically played during corner kicks, goalkeeper kickouts, or long-distance passes, were not performed in this study.

Video analysis

Athletes were monitored by a video system during both training units. Video analysis was used to cross-check the number of ball headings for each player in specific parts of the training.

Blood collection and processing

The blood collection was performed by professional medical personnel at 3 time points: before training, 1 h, and 24 h after each training session. Venous blood was collected in a nonfasting state in K2EDTA tubes (Greiner Bio-One, Austria) and placed on ice until processed. All blood samples were centrifuged within 60 minutes from the time of

blood draw, at 2000 x *g* for 10 minutes at 4°C, and plasma was isolated. Prepared aliquots were stored at -80°C until assayed.

Biochemical procedures

Total tau concentrations in plasma were measured by digital ELISA using the Simoa Tau Advantage Kit (Quanterix, Cat.No. 101552). Tau protein phosphorylated at Thr181 was quantified using the Simoa pTau-181 V2 Advantage Kit (Quanterix, Cat. No. 103714). The samples were blinded prior to analysis and all assays were measured on a HD-X Analyzer (Quanterix Corp, MA, USA). The average coefficients of variation of measurement of Tau and pT181 were 3% and 4%, respectively.

Psychological testing

To monitor cognitive changes after soccer and heading training the Trail Making Test (TMT) was used [40]. The testing consisted of two parts: part A focused on intensity and sustaining of attention; part B focused on cognitive flexibility. All participants were tested before and after completing the training units, at timepoints aligned with the blood sample collection. In the Trail Making Test A, a subject was instructed to connect a set of 25 numbered dots in ascending order (1-2-3-4 etc.) as quickly as possible while maintaining accuracy. In a Trail Making Test B, a subject was instructed to connect the dots in sequence while alternating numbers and letters (1-A-2-B-3-C etc.). Time needed to complete the test was recorded and the results are displayed in seconds (s). Shorter time represents a better score.

© 2023 Cente M et al. *JAMA Network Open.*

eReferences

34. Liu J, Hei C, Luo M, Yang D, Sun C, Feng A. A Study on Impact Force Detection Method Based on Piezoelectric Sensing. *Sensors (Basel)*. Jul 10 2022;22(14)doi:10.3390/s22145167
35. Koizumi A, Hong SC, Sakamoto K, Sasaki R, Asai T. A study of impact force on modern soccer balls. *Procedia Engineer*. 2014;72:423-428. doi:10.1016/j.proeng.2014.06.074
36. Auger J, Markel J, Pecoski DD, et al. Factors affecting peak impact force during soccer headers and implications for the mitigation of head injuries. *PLoS One*. 2020;15(10):e0240162. doi:10.1371/journal.pone.0240162
37. The International Football Association Board. Laws of the Game 2020/2021. https://digitalhub.fifa.com/m/5371a6dcc42fbb44/original/d6g1medsi8jrrd3e4imp-pdf.pdf
38. Queen RM, Weinhold PS, Kirkendall DT, Yu B. Theoretical study of the effect of ball properties on impact force in soccer heading. *Med Sci Sports Exerc*. Dec 2003;35(12):2069-76. doi:10.1249/01.MSS.0000099081.20125.A5
39. Rada A, Kuvacic G, De Giorgio A, et al. The ball kicking speed: A new, efficient performance indicator in youth soccer. *PLoS One*. 2019;14(5):e0217101. doi:10.1371/journal.pone.0217101
40. Reitan RM. Trail making test. Manual for administration, scoring, and interpretation. *Indianapolis, IN: Indiana University Press*. 1956;

© 2023 Cente M et al. *JAMA Network Open.*
