# Supplementary material for: Association of Nonconcussive Repetitive Head Impacts and Intense Physical Activity With Levels of Phosphorylated Tau181 and Total Tau in Plasma of Young Elite Soccer Players
Source: JAMA Netw Open. 2023 Mar 30;6(3):e236101. doi: 10.1001/jamanetworkopen.2023.6101 (PMC10064253; doi:10.1001/jamanetworkopen.2023.6101)
Supplement: Supplement 2. — Data Sharing Statement [file jamanetwopen-e236101-s002.pdf]

Data Sharing Statement

Cente. Association of Nonconcussive Repetitive Head Impacts and Intense Physical Activity With Levels of Phosphorylated Tau_{181} and total Tau in Plasma of Young Elite Soccer Players. *JAMA Netw Open*. Published March 30, 2023. doi:10.1001/jamanetworkopen.2023.6101

Data
Data available: Yes
Data types: Deidentified participant data
How to access data: peter.filipcik@savba.sk; martin.cente@savba.sk
When available: With publication

Supporting Documents
Document types: Statistical/analytic code, Informed consent form
How to access documents: peter.filipcik@savba.sk; martin.cente@savba.sk
When available: With publication

Additional Information
Who can access the data: researchers whose proposed use of the data has been approved
Types of analyses: for any purpose
Mechanisms of data availability: with a signed data access agreement
